# Supplementary material for: Elite Cacao Clonal Cultivars with Diverse Genetic Structure, High Potential of Production, and Good Organoleptic Quality Are Helping to Rebuild the Cocoa Industry in Brazil
Source: Int J Mol Sci. 2025 Apr 4;26(7):3386. doi: 10.3390/ijms26073386 (PMC11989740; doi:10.3390/ijms26073386)
Supplement: Supplementary file 1 [file ijms-26-03386-s001.zip › ijms-3495223-supplementary.pdf]

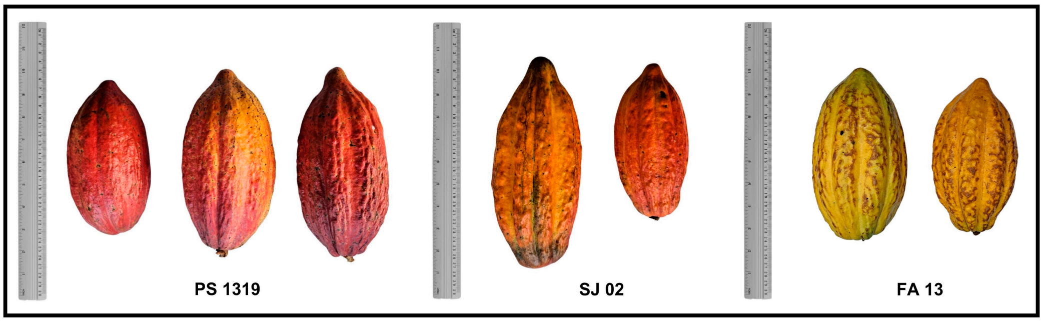

Figure S1. Fruits of Brazilian cacao clonal cultivars.

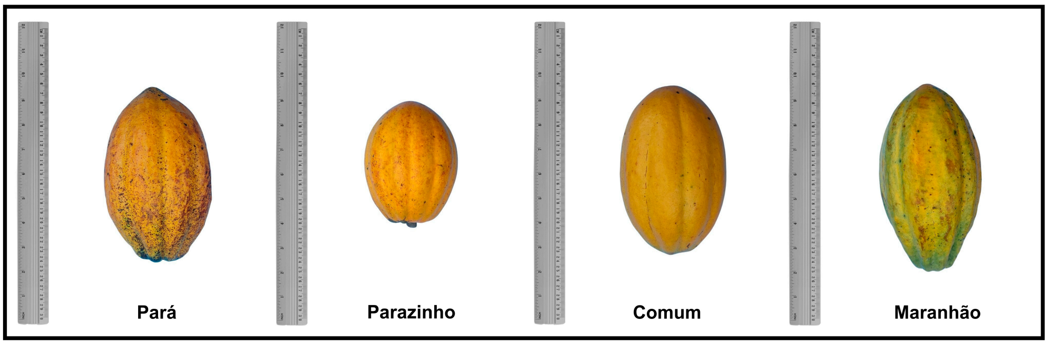

Figure S2. Fruits of Brazilian cacao local cultivars.
